# Pediatric and adult point of view on the gut-kidney axis in CKD

**DOI:** 10.1007/s00467-025-06780-8

**Published:** 2025-07-07

**Authors:** Johannes Holle, Felix Behrens, Laetitia Koppe

**Affiliations:** 1 Department of Pediatric Gastroenterology, Nephrology and Metabolic Diseases, Charité – Universitätsmedizin Berlin, Berlin, Germany; 2 Experimental and Clinical Research Center, a cooperation of Charité – Universitätsmedizin Berlin and Max Delbrück Center for Molecular Medicine, Berlin, Germany; 3 German Centre for Cardiovascular Research (DZHK), Partner Site Berlin, Berlin, Germany; 4 Max Delbrück Center for Molecular Medicine in the Helmholtz Association, Berlin, Germany; 5 Department of General Pediatrics and Hematology/Oncology, University Children’s Hospital, University Hospital Tübingen, Tübingen, Germany; 6 CarMeN Laboratory, INSERM, INRAE, Claude Bernard Lyon 1 University, Pierre-Bénite, France; 7 Department of Nephrology and Nutrition, Hospices Civils de Lyon, Centre Hospitalier Lyon-Sud, Pierre-Bénite, France

**Keywords:** Chronic kidney disease, Gut microbiota, Uremic toxins, Growth, Pediatric

## Abstract

Chronic non-communicable diseases pose a significant global health challenge, with the human gut microbiota emerging as a key player in several (patho) physiological functions, including immunity, metabolic homeostasis, and inflammation. While dysbiosis, or imbalance in taxonomy and function of gut microbiota, has been implicated in chronic kidney disease (CKD), whether it is a cause or consequence of the disease remains controversial. Understanding the gut microbiota’s role in CKD pathogenesis is essential for developing novel therapeutic interventions. CKD in children presents unique opportunities for studying disease-specific mechanisms due to the absence of comorbidities typically seen in adults, such as diabetes, obesity, and hypertension, although few studies exist. On the other hand, unlike the relatively stable gut microbiota of healthy adults, the infant’s microbiome undergoes significant development and maturation during the early years of life. Integrating knowledge from both pediatric and adult populations may provide a comprehensive understanding of gut microbiota dysbiosis in CKD. This review aims to provide an overview of the gut microbiota’s development in healthy individuals and CKD patients and discusses how these findings can inform personalized treatment approaches to CKD.

**Graphical Abstract:**

**Figure:**
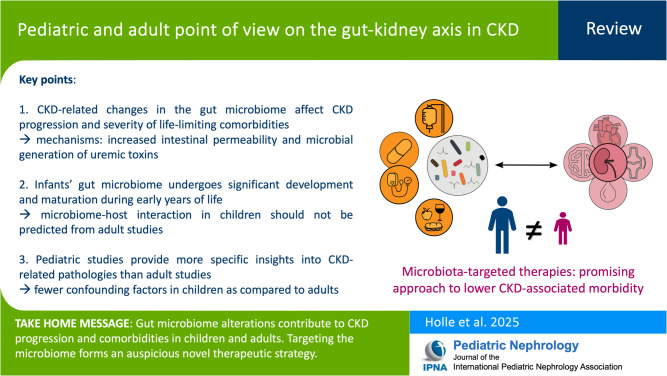
A higher resolution version of the Graphical abstract is available as Supplementary information

**Supplementary Information:**

The online version contains supplementary material available at 10.1007/s00467-025-06780-8.

## Introduction

Chronic non-communicable diseases are the leading cause of mortality in the world and pose a great threat to human health [1]. The human gut microbiota is a complex and dynamic ecosystem that naturally lives in a symbiotic relationship with the host. The commensal inhabitants of the gut are as numerous as they are diverse: bacteria, viruses, archaea, and eukaryotes together comprise the gut microbiome as 10^14^ microbes. Among these, Bacillota (e.g., *Faecalibacterium*, *Roseburia*, *Ruminococcus*, *Eubacterium*) and Bacteroidota (e.g., *Bacteroides*, *Alistipes*, *Prevotella*) dominate, followed by Actinomycetota (e.g., *Bifidobacterium*, *Collinsella*) and Pseudomonadota (e.g., *Escherichia*). These are accompanied to a much lesser extent by other phyla such as Synergistota, Verrucomicrobiota, Fusobacteriota, and Methanobacteriota [2–4].

Over the last decade, the gut microbiota has emerged as a key regulator of several physiological functions such as immunity, metabolic homeostasis, and inflammation [5–7]. Knowledge of the role of the gut microbiota, its development, and host-microbial interactions in human health and disease has rapidly increased due to the advancement of modern molecular technologies. In particular, the standardization of sampling methods, the depth of genomic analysis, and the increasing biostatistical power of analyses have enabled us to describe the composition of the microbiota in more detail and understand more of its functions [8].

As in other chronic diseases, numerous studies in chronic kidney disease (CKD) have focused on the communication between microbes and the host [9]. However, the role of dysbiosis in the progression and cause of CKD is controversial [10, 11]. Whether dysbiosis itself is one of the initial causes or the consequence of CKD remains a topic of debate. The majority of studies are observational, limiting the ability to demonstrate causality and thus constraining therapeutic options. It is difficult to distinguish between features that are purely attributable to CKD and those that may be biased by environmental, dietary, and comorbidity-associated factors. To address the question of causality, most studies have been based mainly on experimental data. The use of fecal microbiota transfer (FMT) from CKD donors into germ-free animals may provide partial answers to this question [12, 13]. However, to translate these findings to humans and develop appropriate therapies, clinical studies are warranted.

In adults, CKD is mainly driven by diabetes and arterial hypertension, which could influence the gut microbiota composition and complicate the interpretation of specific CKD gut microbiota signatures. These comorbidities are usually absent in children with CKD. Thus, in the absence of confounding classical risk factors, dysbiosis is expected to be induced largely by CKD-specific mechanisms. In contrast to the mature intestinal microbiota of healthy adults, which appear relatively stable over time, the infant’s microbiome only establishes and matures during the first years of life. Therefore, combining our knowledge of the microbiota in children and adults with CKD could be an opportunity to highlight microbiota signatures specific to CKD and potentially identify therapeutic targets independent of confounding factors.

The aim of this review is multifaceted: first, to provide an overview of key differences of gut microbiota development in healthy life as compared to individuals with CKD, both in adult and pediatric stages. Of note, we aim to emphasize the role of host-related and environmental factors as potential confounding variables and describe quality standards for microbiota analysis. Second, we explore how these insights can inform the identification of new therapeutic targets, particularly by clarifying the potential role of gut microbiota in CKD-related pathophysiology.

## The healthy gut microbiome across the lifespan

**Fig. 1:**
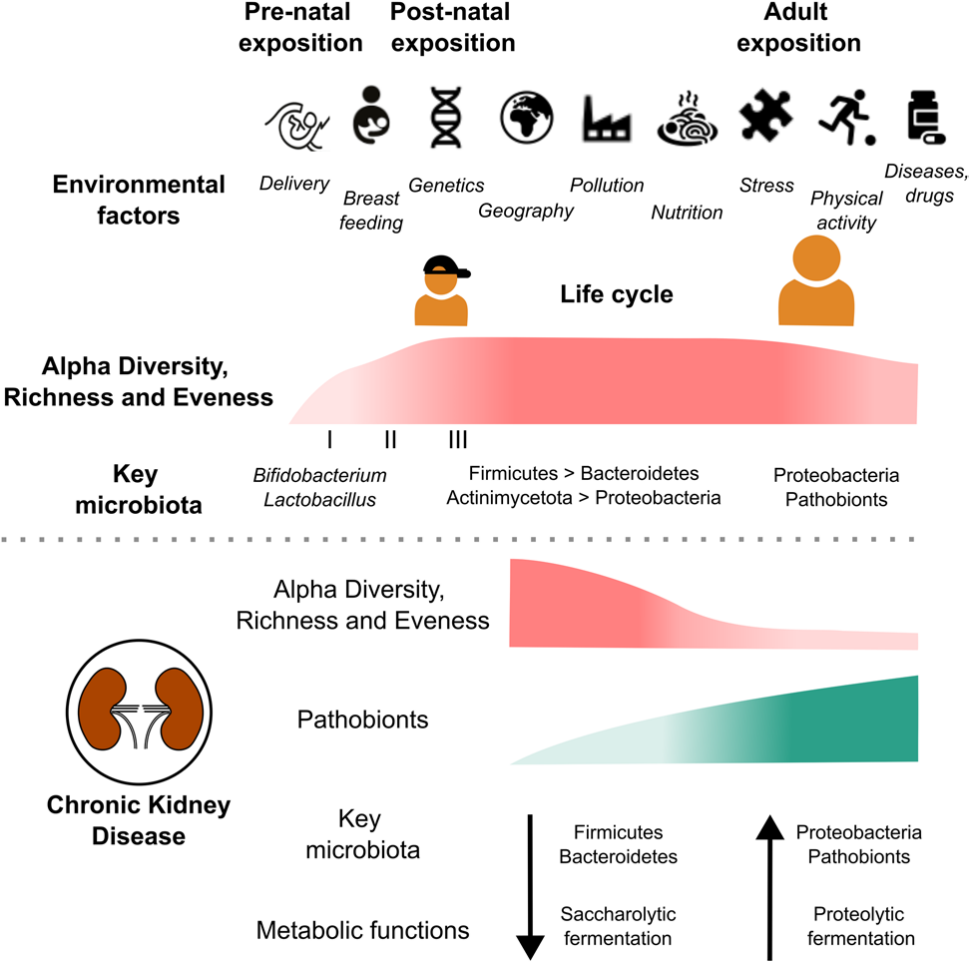
Development of the gut microbiome in health and CKD. The developing gut microbiome undergoes distinct phases in early childhood (adapted from Stewart et al. [16]). The developmental (I, 3–14 months) and transitional phase (II, 15–30 months) are characterized by an increase in microbiome diversity and richness with key microbiota developing in response to dietary modifications end environmental interactions. By the age of 30 months, the composition of the microbiome remains relatively stable over decades in healthy individuals (III, > 30 months). Irrespective of the time of onset, CKD is characterized by a reduction in alpha diversity developing side by side with a loss of saccharolytic bacteria and a bloom of Proteobacteria and pathobionts

Aging significantly influences gut microbial communities, and recent research on the evolution of the gut microbiota across large populations, from newborns to elderly individuals, has highlighted various trajectories of the microbiota throughout the human lifespan (Fig. 1). The functions of the gut microbiome significantly impact childhood development. In contrast to the relatively stable intestinal microbiota found in healthy adults, an infant’s microbiome evolves and matures throughout the early years of life [14, 15]. Therefore, understanding the mechanisms that establish and sustain a healthy microbiota composition is crucial. This is particularly important during the immediate postnatal and early infant periods when initial bacterial colonization occurs and the microbiota undergoes dynamic changes.

Of note, there is an ongoing discussion about when exactly first microbial colonization occurs. Although many studies have reported gut microbial colonization before birth, more recent investigations convincingly demonstrated that gut colonization does not occur in healthy fetuses before birth [17, 18]. The previous reports are likely the result of contamination between sample collection and processing, illustrating common pitfalls in the microbial analysis of low-biomass specimens [19].

The early development and maturation of the gut microbiota are highly dynamic processes influenced by various perinatal conditions, including external factors (e.g., mode of delivery, type of feeding, antibiotic use, lifestyle, and geographic factors) and host factors [20]. The majority of studies have focused on the human gut microbiota in individuals from 0 to 3 years of age and in adulthood and the elderly, but very few in children from 3 to 18 years old, possibly due to difficulties obtaining samples commonly linked to ethical/practical issues particularly around puberty [21, 22]. Based on these difficulties, during the first 3 years of life, the majority of the studies have revealed common patterns of development across different countries [21, 22]. The microbiota develops from the initial inoculum, which is poor in microbial community structure and diversity, to a complex and diverse ecosystem during the first years of life. Over time, additional bacteria are incorporated generating an increasingly diverse bacterial ecosystem with significant resilience to exogenous perturbations [23]. Based on a large study including 903 infants from Germany, Finland, Sweden, and the USA, three distinct phases of gut microbiota development have been identified based on the dynamics of the most abundant phyla (Actinobacteria, Bacteroidetes, Firmicutes, Proteobacteria, and Verrucomicrobia) and changes in alpha diversity (i.e., microbial richness and balance, indicating ecosystem health) [16]. The phases are as follows (Fig. 1): a developmental phase (months 3–14) dominated by *Bifidobacterium* and further characterized by gradual changes in all detected phyla and a steady increase of alpha diversity (richness and Shannon’s diversity), a transitional phase (months 15–30) where only Bacteroidetes (further enrichment) and Proteobacteria (reduction) continue to develop while alpha diversity continues to grow, and a stable phase (31 months and beyond) where both the present phyla and alpha diversity stabilize. During the developmental phase, *Bifidobacterium* spp. dominate, whereas the stable phase features higher bacterial diversity with a predominance of Firmicutes. Even though the microbiota had acquired a configuration similar to that of the adult at the age of 5, it still has a lower community richness and lacks certain essential taxa at 5 years old present in the adult microbiota such as *Methanobrevibacter* and *Christensenellaceae* [24].These late colonizers correlate with increased alpha diversity in both children and adults [24]. It is important to note that these studies also confirm that children have individual dynamics in the gut microbiota development trajectory. All of this suggests that the complete maturation of the gut microbiota may take longer, particularly for certain microbial members. At the other end of the lifespan, most studies on patients over 65 years old have shown a decrease in both alpha and beta diversity (i.e., differences in microbial composition) compared to healthy adults [25]. While differences in taxonomic composition and functional potential varied across studies, *Akkermansia* was consistently found to be relatively more abundant in the elderly, whereas *Faecalibacterium*, *Bacteroidaceae*, and *Lachnospiraceae* were relatively reduced [25]. The cross-sectional design of these studies limits definitive interpretations, highlighting the need for more longitudinal research in this population.

In conclusion, the early postnatal colonization process is vital for establishing the long-term composition of the microbiota, which in turn influences immune system maturation, brain development, and overall body growth [22]. Increasing evidence suggests that alterations in the microbiota during this critical period are linked to negative health outcomes [26]. In particular, based on experiments involving fecal transplantation from young to aged animals [27], it is now evident that gut microbes and their metabolites (some of which can cross the blood–brain barrier) actively contribute to neurodevelopmental processes. These include the establishment of the blood–brain barrier, neurogenesis, microglial maturation, and myelination, profoundly influencing the transcriptional programming of several key brain regions as summarize by Dash et al. [28]. This highlights the importance of understanding the factors that can disrupt this maturation, as a healthy juvenile microbiota could be a promising target for preventing cardiovascular and degenerative diseases [27, 29]. However, there are currently no extensive studies exploring the relationship between gut microbiota dynamics and the incidence of CKD.

## CKD gut microbiota studies

### Gut microbiota composition in adults and children with CKD

For the past 15 years, accumulating studies have consistently shown differences in the microbiome of patients with CKD compared to that of healthy individuals. To illustrate this, we have summarized in Table 1 original studies published in English that primarily focused on alterations in keystone taxa and/or diversity in children and adults with CKD at various stages. Studies were excluded if they lacked a control group without kidney dysfunction or did not report eGFR values (except for patients on dialysis). However, these studies are largely limited by small sample sizes (fewer than 100 patients) and often rely on basic microbiota analysis techniques, such as 16S rRNA sequencing. Furthermore, most do not adhere to recently proposed guidelines designed to ensure accurate analysis and interpretation of microbiota composition [9]. Key requirements include fecal sample-specific parameters, such as the time since the last defecation and the Bristol Stool Form Scale, as well as the standardized collection of metadata, including treatment regimens and dietary habits. Additionally, the adoption of absolute microbiome quantification methods, as described by Vandeputte et al. [30], is strongly encouraged to significantly enhance the interpretability of microbiome changes.

**Table 1:** Summary of kidney disease studies on gut microbiota compared to healthy controls

CKD stage	Study population CKD (control)	Country	Mean age (years)	Mean eGFR (ml/min/1.73 m^2^)	Profiling methods	Alpha diversity	Key gut-microbiota metabolites	Key gut microbiota composition (compared to control)	Ref
**Pediatric studies**									
2–3	26 (60)	Taiwan	13.7	79	16S rRNA	ND	↓Urinary concentrations of TMAO and DMA in CKD stage 2–3	↓Genus *Prevotella* in CKD children with hypertension	[31]
2–4	34 (51)	Taiwan	11.2	100.3	16S rRNA	ND	↑Plasma levels of butyric acid and propionic acid in CKD children with hypertension	↑Phylum Verrucomicrobiota ↑Genus *Akkermansia* ↓Species *Bifidobacterium bifidum* in CKD children with non-glomerulonephritis	[32]
2–4	36 (79)	Taiwan	11.3	100.7	16S rRNA	No change	↑Plasma concentrations of DMA, TMA, and TMAO in children with CKD stage 2–4	↓Phylum Cyanobacteriota ↓Genera *Subdoligranulum*, *Ruminococcus*, *Faecalibacterium*, and *Akkermansia* in CKD children with hypertension	[33]
2–4	28 (77)	Taiwan	9.60	107.10	ND	ND	Higher index plasma propionate was positively correlated with decreasing ejection fraction. The index of higher plasma butyrate was positively correlated with worsened blood pressure at 1-year follow-up	ND	[34]
3–4/5 (HD)	23 (10)	Germany	10.6	29.6*	16S rRNA	No change	Plasma metabolite analysis showed a stage-dependent increase in tryptophan metabolites	↑Proteolytic species (such as *Citrobacter*) ↓Saccharolytic species (such as *Bifidobacterium*)	[35]
5 (KD, PD)	16 (13)	USA	13.6 HD and 11.9 for PD	< 15	16S rRNA	↓α-diversity in PD = α diversity for HD	↑Plasma levels of IS and PCS in HD and PD	↑Phylum Bacteroidota in HD ↓Phyla: Bacillota and Actinomycetota in PD ↑Family *Enterobacteriaceae* in PD	[36]
**Adult studies**									
5 (HD)	24 (12)	USA	57	< 15	16S rRNA	No change	ND	↑Species *Brachybacterium*, *Catenibacterium*, *Enterobacteriaceae*, *Halomonadaceae*, *Moraxellaceae*, *Nesterenkonia*, *Polyangiaceae*, *Pseudomonadaceae*, and *Thiothrix* families	[37]
5 (HD)	53 (69)	China	55.5 for CKD and 54 for HF	5.33	16S rRNA	↓	Concentrations of IS and PCS were correlated with the gut microbiome	↑Species *Neisseria*, *Lachnoclostridium*, and *Bifidobacterium* *↓*Species *Faecalibacterium*	[38]
4, 5	20 (20)	USA	62.8	16.54	16S rRNA	ND	↑LPS	↑Phlya Pseudomonadot*a*, Verrucomicrobiota, and Fusobacteriota	[39]
1–5	110 (210)	China	51.7	37.18	16S rRNA	↓	ND	↑Species: K*lebsiella* and *Enterobacteriaceae* *↓Species: Blautia* and *Roseburia*	[40]
1–5 (PD)	168 (30)	Taiwan	62.4 for mild, 63.6 for moderate, 66.2 for advanced	71.4 for mild, 59.9 for moderate, 16.2 for advanced	16S rRNA	↓	↑Microbial genes related to the metabolism of aromatic amino acids	↑Genera:*Escherichia shigella*, *Dialister*, *Lachnospiraceae_ND3007_group*, *Pseudobutyrivibrio*, *Roseburia*, *Paraprevotella*, and *Ruminiclostridium* ↑Species: *Collinsella stercoris* and *Bacteroides eggerthii*	[41]
1–5	72 (20)	Taiwan	62.6 for mild, 64.4 for moderate, 65.0 for advanced	74.4 for mild, 52.3 for moderate, 16.6 for advanced	Shotgun	↓	6 circulating metabolites (among these ↑PCS) were significantly altered across early to advanced stages	13 microbial species (among these *↓ Prevotella* sp. *885*) were significantly altered across early to advanced stages	[42]
3–5	80 (78)	China	49.50	21.0	16S rRNA	↓	No difference in SCFA levels in feces	Phylum: ↑Bacteroidota and *↓*F Fusobacteriota Genus: *↓Lachnospira*, *Roseburia*, *Megamonas*, *Megasphaera*, *Fusobacterium*, and *Akkermansia* Genus: ↑*Parabacteroides*, *Oscillospira*, and *Ruminococcus*	[43]
5 (HD)	223 (69)	China	53.0	< 15	Shotgun	ND	A group of microbial species enriched in HD encode functions involved in toxin and secondary bile acids synthesis; the relative abundance of the microbial functions correlates with the serum or fecal concentrations of these metabolites	↑Species *Eggerthella lenta*, *Flavonifractor* spp. (mainly *F. plautii*), *Alistipes* spp. (mainly *A. finegoldii* and *A. shahii*), *Ruminococcus* spp., and *Fusobacterium* and *Fusobacterium nucleatum* *↓*Species *Prevotella* spp. (mainly *P. copri*), *Clostridium* spp. and several butyrate producers (*Roseburia* spp., *Faecalibacterium prausnitzii*, and *Eubacterium rectale*)	[12]
3–5	25 (34)	China	51.08	ND	16S rRNA	No change	ND	↑Phylum Pseudomonadota *↓*Phylum Bacteroidota	[44]
1–5	100 (100)	China	56.7	56.9	16S rRNA	↓	↑Metabolic pathways of bacterial toxin, chloroalkane and chloroalkene degradation	↑Species: *Actinobacteria*, *Alistipes*, *Bifidobacterium*, and *Bifidobacterium longum*	[45]
3	37 (74)	Japan	68.8	53.7	Shotgun	No change	ND	*↓*Butyrate-producing species *Roseburia inulinivorans*, *Ruminococcus torques*, and *Ruminococcus lactari* ↑Species *Bacteroides caccae* and *Bacteroides coprocora*	[46]
2–4	72 (20)	Taiwan	63.9	59.9	Shotgun	↓	Correlation analyses among systems-level microbiome, serum metabolome and immune parameters revealed coordinated host–microbe relationships in CKD, including a role of gut-derived tryptophan metabolism in B cell immunity during kidney impairment	↑Abundance of carbohydrate active enzyme (CAZyme) genes in gut	[47]

*16 sRNA* 16S ribosomal RNA, *CKD* chronic kidney disease, *DMA* dimethylamine, *eGFR* estimated glomerular filtration rate, *HD* hemodialysis, *IS* indoxyl sulfate, *LPS* lipopolysaccharide, *ND* no data, *PCS* p-cresyl sulfate, *PD* peritoneal dialysis, *SCFA* short-chain fatty acids, *TMAO* trimethylamine N-oxide

Many studies observed a decrease in alpha diversity compared to healthy controls (Fig. 1). Almost all studies highlighted a difference in beta diversity, showing distinct bacterial compositions in CKD as compared to healthy controls [9, 48]. In terms of taxonomy, an increased abundance of the phyla Proteobacteria and Fusobacteria and the genera *Escherichia Shigella*, *Desulfovibrio*, and *Streptococcus*, along with a lower abundance of the genera *Roseburia*, *Faecalibacterium*, *Pyramidobacter*, and *Prevotella*, have been observed frequently in adult patients with CKD. In children, although the number of studies is even more limited, the majority observed an increase in the phylum Proteobacteria and genera *Bacteroidaceae* and a decrease in the genera *Faecalibacterium* and *Bifidobacterium* [49].

Based on these studies however, it is difficult to draw conclusions on specific gut microbiota modifications caused by CKD due to large variation in patient age at stool sample collection, different microbiological profiling methods, and inadequate control for potential confounders such as diet, physical activity, medical treatment, and comorbidities [49]. Also, most of the studies relied on amplicon sequencing that can characterize the gut microbiota at the genus level only and had limited sample size (< 100), primarily focusing on patients with kidney failure.

Although the differences in CKD patients may distinguish patients from heathy individuals, its usefulness as a biomarker has yet to be demonstrated. One study has shown that gut microbiota can discriminate cases of different severities from controls. These strategies were proven in a cohort of 110 patients, using a control cohort and external validation [40]. However, while the KFRE (Kidney Failure Risk Equation) is clearly more powerful in discriminating severity, the key question is if microbiota composition may predict progression of CKD or CKD-related comorbidities, which would provide additional value to classical markers of kidney function. Until now however, no studies have addressed this issue either in adult or pediatric patients, and future studies would have to consider the large inter-individual and geographical variability of the microbiome.

### Gut-derived metabolites in adults and children with CKD

The gut bacterial community plays a crucial role in regulating various aspects of metabolic disorders and subsequently also immunity through the production of a diverse array of metabolites. These bacterial metabolites range from small molecules to large macromolecules and include byproducts of bacterial metabolism, such as short-chain fatty acids (SCFAs) and molecules derived from aromatic amino acids (e.g., phenol and indole) or choline (trimethylamine N-oxide, TMAO). Additionally, complex macromolecules essential for bacterial integrity, such as peptidoglycan and lipopolysaccharides (LPS), are involved in these interactions [50–52].

Molecules typically excreted by healthy kidneys into the urine but retained in the body due to impaired kidney function are referred to as uremic retention molecules. Among these, those that induce detrimental clinical, biochemical, or biological effects are classified as uremic toxins. Across studies, the progression of CKD has consistently been linked to significant changes in gut-derived compounds, characterized by an accumulation of indoles, phenols, and TMAO (thus fitting the definition of uremic toxins), along with a reduction in SCFAs [53]. However, the serum concentration of these metabolites may not only reflect gut production. High levels of gut-derived compounds may also result from reduced kidney clearance and increased intestinal permeability [54]. There are discrepancies regarding the specific contribution of gut microbiota to the significant increase in plasma uremic toxin levels. Gryp and colleagues [10] did not observe an increase in uremic precursor levels in fecal samples from CKD patients compared to healthy individuals, concluding that the elevation in plasma uremic toxins concentrations is largely due to decreased kidney function. Conversely, Wang and colleagues found a correlation in CKD patients between fecal uremic toxins precursors and plasma uremic toxins concentrations, as well as the abundance of genes encoding uremic toxin-synthesizing enzymes in the gut microbiota [12]. These differences may be attributed to varying methodological approaches but also highlight that both increased metabolite production and decreased kidney clearance are likely to contribute to the accumulation of detrimental microbial metabolites in CKD. Although data are very limited for children, a recent publication supports the role of enhanced microbial production in the accumulation of the uremic toxin TMAO [55].

Additionally, the presence of genes in the microbiota does not consider post-translational modifications. For example, sulfation of gut microbiota tryptophanase could reduce the activity of these enzymes [56]. Therefore, global studies combining dynamic and deep functional analysis of the gut are needed to determine the influence of the gut in all adults and children with CKD. By “dynamic analysis,” we refer to in vitro systems that facilitate real-time measurement of microbial activity, interactions under varying conditions, and the effects of post-translational modifications. “Deep functional analysis” entails detailed investigations using shotgun metagenomic approaches to examine microbial genes, pathways, and their functions, providing a comprehensive understanding of their roles in disease.

Another question is whether age influences plasma gut microbiota-derived metabolite concentrations, which is difficult to investigate. Substantial differences can be expected compared to adults, since children have larger body water volumes and lower circulating proteins, higher protein and caloric needs per kilogram of body weight, a different pattern of underlying kidney disease, and maturational changes in organic solute transport at the proximal tubule, along with significant modifications of gut microbiota [57]. To investigate this, in a large study of children with CKD (*n* = 609) and on hemodialysis (*n* = 170) [58], age was found to contribute to plasma-bound uremic toxins (PBUTs) such as p-cresyl sulfate (PCS) and indoxyl sulfate (IS) levels, as previously demonstrated in the adult CKD population [59]. Remarkably, while age-dependency of IS and PCS was also found in healthy adults, no positive correlation between age and PBUTs in healthy children was found, suggesting a different basis of age-dependency of PBUTs in adults versus children [60, 61]. Therefore, it is unlikely that the distribution, inter-compartmental clearance, removal pattern during dialysis, and retention profile of uremic toxins would be identical to those in adults [57]. All of this confirms that we cannot simply transpose data on the distribution of uremic toxins from adults to children, and specific studies are needed.

### The gut microbiome as a modifiable risk factor and treatment target in CKD

Microbial metabolites are known to associate with comorbidities in CKD, such as cardiovascular disease [58, 62]. Animal models have established a functional relationship between accumulated metabolites (IS, PCS, TMAO) and cardiovascular disease [63–65], suggesting that modulating the microbiome in CKD could be a promising therapeutic target to dampen not only CKD progression but also the burden of frequent and life-limiting comorbidities.

**Fig. 2:**
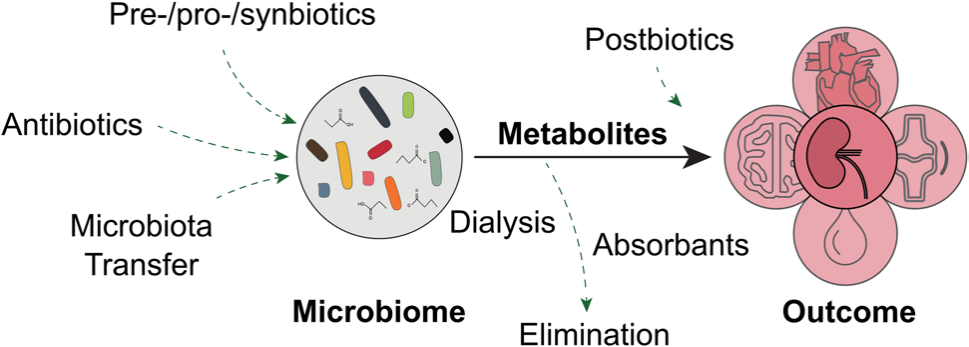
Microbiome-targeting therapies to attenuate progression of CKD-related comorbidities. Various therapeutic interventions have been investigated or are under development to modulate the composition and the functional properties of the gut microbiota in CKD (including antibiotics, pre-/pro- and synbiotics as well as microbiota transfer). In addition, the clearance of toxic metabolites, which accumulate because of impaired kidney function by intensifying dialysis or reducing the uptake, as well as the supplementation of beneficial metabolites (postbiotics) are promising strategies

Looking at clinically established procedures, such interventions (summarized in Fig. 2) include dietary changes [66], such as high fiber diet, but also more specific strategies, i.e., prebiotics (nonviable dietary substances that modulate the microbiota) [67], probiotics (live microorganisms) [68, 69], postbiotics (bacterial metabolites) [70], and synbiotics (a combination of prebiotics and probiotics) [71]. Numerous proof-of-concept studies attest to the potential benefits of probiotics and prebiotics consumption in modulating the gut microbiota of CKD patients [67, 69, 72]. However, a recent meta-analysis found limited evidence supporting biotic supplementation in adult CKD management, likely due to the empirical selection of both prebiotic and probiotic strains, guided by their effects in other diseases or the use of available strains, rather than systematic scientific investigation [73]. The key question is how to select appropriate strains to limit kidney function degradation.

Studies consistently demonstrate that the survival, safety, and efficacy of probiotic candidates are both strain-specific and disease-specific, meaning their benefits cannot be generalized [67, 69, 72, 73]. For instance, strains with urease activity can increase the production of NH_3_/NH_4_OH, which damages epithelial tight junctions and may allow endotoxins and uremic toxins to enter the bloodstream. To address such challenges, regulatory agencies have established guidelines for evaluating new probiotic or synbiotic candidates. These guidelines incorporate in silico, ex vivo, and in vivo studies, with genomics serving as a key tool for rapid screening [74]. However, these advanced approaches have not yet been applied in the context of CKD. In the meantime, other approaches have been proposed. For example, Li et al. reported that *Faecalibacterium prausnitzii* depletion in CKD patients was associated with impaired kidney function, and supplementation improved outcomes [75]. Similarly, Zhu et al. demonstrated that *Lactobacillus casei Zhang*, selected for its immune-modulating properties, enhanced SCFA levels and improved kidney function in CKD patients [76]. These findings highlight the need for a more rigorous and standardized process in probiotic research, particularly for CKD, to ensure the effective selection and application of strains. Also, considering the high inter-individual differences in the microbiota, one could speculate that designing individual combinations of pre- and probiotics may be more effective. However, reliable biomarkers to select individual regimens are still lacking and will be of interest in future studies. For children with CKD, it remains to be elucidated whether microbiome-targeted approaches are effective in pediatric CKD given the lack of rigorous study [77].

An alternative approach focuses on targeting specific gut bacterial enzymes with inhibitors. For instance, the small molecule 3,3-dimethyl-1-butanol (DMB), which mimics the structure of choline, functions as a potent inhibitor of TMA lyase. By blocking the activity of TMA lyase, DMB significantly reduces the production of TMA and subsequently the formation of the deleterious metabolite TMAO [78].

In experimental animal and preliminary clinical studies, other methods to influence the microbiota or the systemic concentrations of bacterial metabolites show promising results. These include deeper manipulation of the microbiome by antibiotic depletion, as carried out in many animal models [79] and in small cohorts of patients with kidney failure [80], or complete transfer of a different microbiome by FMT. Experimental data suggest that FMT is efficient to reduce uremic toxins production but the clinical impact must be demonstrated in patients with CKD [13, 81]. Some recent studies are encouraging, as it was recently shown that FMT promotes tolerance to stem cell transplantation by induction of regulatory immunity [82]—an effect that might also be beneficial in CKD to lower the established inflammatory burden that is known to contribute to disease progression and comorbidities [83].

In another approach, spherical carbon adsorbent (e.g., AST- 120) was administered orally to patients with CKD to lower the intestinal uptake of uremic toxins. Although there were no serious side effects, strong evidence on the effectiveness of oral adsorbents is still lacking and has not been tested in the pediatric population [84]. Further improvements of this approach are on the way, mainly aiming at a more selective and effective absorption of uremic toxin precursors in the gut [85]. As most of the circulating uremic toxins are bound to plasma proteins, the removal of PBUT remains challenging [86], but could be improved by novel adsorption-based hemodialysis technologies [87].

Taken together, these interventions might extend the set of tools to modulate the gut microbiota and uremic toxin burden and its effects on the host and could significantly enhance patient care in the future. Until then, however, thorough large-scale clinical studies of patients with CKD of multiple age ranges will have to be carried out to test their feasibility, safety and efficacy.

## Gut microbiome and CKD: lessons to be learned for children and adults

### Gut-kidney axis across the lifespan—changes in the microbiome as a predictor of long-term kidney outcome?

Much of our knowledge about the structure, function, and dynamics of the human gut microbiome is generally based on cross-sectional or short-term longitudinal studies [88]. It was evidenced, however, that insults early in life can lead to kidney reprogramming that is known to contribute to CKD development later in life [89]. Various hypotheses were proposed to explain this phenomenon, including the thrifty phenotype hypothesis, predictive adaptive responses, and the catch-up growth hypothesis [90]. After birth, the development of the microbiome is highly diverse and depends on a plethora of factors that include, amongst others, nutritional factors, such as breastfeeding [91]. Recent data suggest that early lifestyle, medical conditions, and diet impact the microbiome not only for a short period of time but rather shape the microbiome for decades, if not even the rest of life [92]. It is, hence, tempting to speculate whether early alterations of the microbiome may contribute to the development of chronic diseases associated with dysbiotic microbiota, especially CKD, later in life. Notwithstanding, longitudinal studies backing such associations are lacking and will also have to be supplemented by mechanistic analyses.

### Exploring the causality further: the egg or the chicken?

**Fig. 3:**
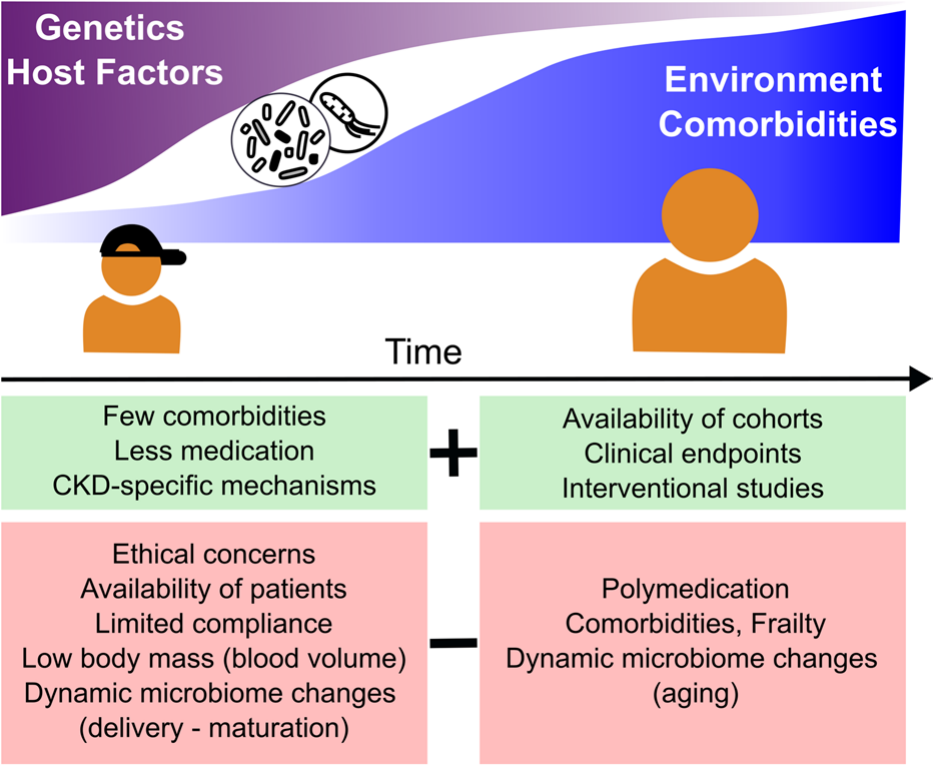
Pediatric and adult studies on CKD. The fundamental differences between pediatric and adult CKD studies are summarized, illustrating the dominant role of host and genetic factors in pediatric CKD in comparison to environmental factors and comorbidities characterizing adult CKD. Specific advantages, potential pitfalls, and obstacles are highlighted for studies in both pediatric and adult CKD patients

Both adult patients and children with CKD present different advantages and limitations for studying the direct impact of the disease on gut microbiota, as summarized in Fig. 3. Growth and puberty, unique to pediatric patients, significantly influence gut microbiota composition. While CKD in children is primarily due to congenital anomalies of the kidney and urinary tract (CAKUT) and hereditary kidney diseases [93], kidney failure in adults is mainly caused by conditions such as glomerulopathies (e.g., diabetic nephropathy, hypertension) and autosomal dominant polycystic kidney disease [1], with hypertension and diabetes also strongly influencing the gut microbiota [94]. Given that kidney organic anion transporters play a critical role in determining PBUTs plasma levels in CKD, tubulopathies could strongly modify uremic toxin metabolism and may, therefore, limit applicability of results from affected patients to patients with other underlying primary condition [95].

By combining a comparative approach (between “young” and “old”) that balances the limitations of both populations, we may determine which key bacterial taxa prevent or promote disease onset. For example, patients with CKD (both children and adults) demonstrate a noticeable reduction in the abundance of bacteria responsible for generating SCFAs, such as *Faecalibacterium prausnitzii*, which is associated with low fecal concentrations of butyrate [75, 96]. SCFAs are instrumental in maintaining intestinal mucosal integrity, regulating metabolism, controlling energy expenditure, and modulating the immune system [97]. These data suggest that these bacteria, or their metabolites (such as butyrate), could be promising therapeutic targets in CKD, as suggested by preclinical data, where *Faecalibacterium prausnitzii* and butyrate improve kidney function [75, 76, 98].

So far, most studies focus on the impact of the microbiome on the host. It may, however, be short-sighted to omit the bi-directionality of microbiota-host interaction and recent data pinpoint that host factors, and not only exogenous determinants like diet, medication and environment, also shape the microbiome. In CKD, the accumulation of urea was proposed to contribute to the altered proteolysis-skewed fermentation pattern that favors the production of gut-derived uremic toxins by increasing the intestinal pH [54, 77, 99]. Moreover, inflammation and cardiovascular events, potentially also via inflammatory activation, were shown to significantly alter the microbiome. Experimental myocardial infarction in mice led to a depletion of *Lactobacillus* and *Prevotella* [100]—features also known in CKD patients [62, 77]. Beyond compositional changes, it was demonstrated that loss of the cytokines IL-22 or IL-23 propels TMAO production in mice and can thereby aggravate the cardiovascular phenotype [101]. Considering that both inflammation and cardiovascular disease are highly prevalent in CKD, future studies will have to delineate if these findings are also applicable in CKD and may pave the way to a deeper understanding of dysbiosis development in CKD and subsequent identification of novel therapeutic targets.

### Improving and refining our main objectives for gut microbiota research

Microbiome research forms a rapidly growing field and has provided many compelling and insightful studies in recent years. Notwithstanding, the complexity of microbiome-host interactions has left many gaps of knowledge thus far and especially obscures the identification of specific mechanisms impacting how the microbiome impacts host health. Moreover, the primary endpoints of studies in children and adults with CKD differ significantly. The low mortality rates in children with CKD [102] make using mortality as a primary endpoint in pediatric studies less relevant or at least insufficient. In contrast, endpoints such as neurocognition, brain development, and growth, which are less studied in adults, are particularly relevant for children. Based on these observations, several studies in recent years have explored the association between plasma gut-derived metabolites and patient-centered outcomes (i.e., quality of life, symptom severity, and treatment satisfaction) in patients CKD [103–105], but more research is needed, particularly regarding psychological outcomes. Similar studies should be conducted to explore the association between gut microbiota composition and psychological aspects.

In the adult population, there is increasing knowledge about the relevant contribution of microbial dysbiosis on health and disease [106, 107]. In line with the high incidence and prevalence of CKD and CKD-related comorbidities in adults, interventional studies need to address hard clinical endpoints in addition to reporting of beneficial effects of microbiome-centered therapies on the host metabolism [108]. On the other hand, studies in pediatric patients might be more effective in deciphering the CKD-specific interplay between the gut microbiome and host metabolism and immunity, as confounding comorbidities are usually absent. The assessment of early cardiovascular damage, for instance carotid intima-media thickness and pulse wave velocity, can provide additional information on the CKD-specific toxicity on the cardiovascular systems in children with CKD [58].

Concerning growth, it is now widely accepted that disturbances in gut microbiota development, particularly during the first 2 years of life, can affect growth trajectories [109]. The absence of gut microbiota or dysbiosis negatively impacts circulating levels of Insulin-like Growth Factor 1 (IGF-1) in germ-free mice [110]. Microbial stimulation supports the activity of the somatotropic axis in juveniles by improving the growth hormone (GH) sensitivity of peripheral tissues and increasing circulating levels of IGF-1. In CKD, a state of GH/IGF-1 resistance is well known and primarily results in growth disorders in children [110]. However, consequences of GH/IGF-1 resistance in adults with CKD are equally severe, leading to muscle atrophy, protein energy wasting, and lower bone density, all correlated with cardiovascular events and mortality, underscoring its central role in CKD pathology [111, 112]. However, the role of gut microbiota in this pathophysiological pathway was not studied in adults until now. Therefore, a better understanding of the role of gut microbiota in GH/IGF-1 resistance in children could help maintain nutritional status and regular growth in this specific population and should be translated to adults with CKD to investigate its potential importance for severe CKD-associated comorbidities.

## Conclusion

This review highlights a significant gap in knowledge regarding the intestinal microbiota in CKD, particularly in the pediatric population, where studies are often limited by small sample sizes and a lack of depth in analyses and bioinformatics tools. This insufficient understanding has resulted in the empirical use of therapeutic strategies to modulate the microbiota. It is important to note, as children have several peculiarities, the translation of adult knowledge on uremic toxic production and gut microbiota composition to childhood might be skewed. On the other hand, we are convinced that gut microbiota research in the pediatric population comprises additive value in clinically elucidating the intrinsic toxicity of gut microbiota for all CKD patients including adults and the elderly. Subsequently, better understanding of gut microbiota toxicity by gut microbiota-derived metabolites offers a robust pathophysiological underpinning for the development of novel dietary and pharmacological interventions, with the goal not only to improve the health of patients with CKD but, hopefully, also prevent its progression to kidney failure.

## Supplementary Information

Below is the link to the electronic supplementary material.

Graphical abstract (PNG 981 KB)
