# Supplementary figures and images for: Pediatric and adult point of view on the gut-kidney axis in CKD
Source: Pediatr Nephrol. 2025 Jul 7;41(3):633–48. doi: 10.1007/s00467-025-06780-8 (PMC12852165; doi:10.1007/s00467-025-06780-8)

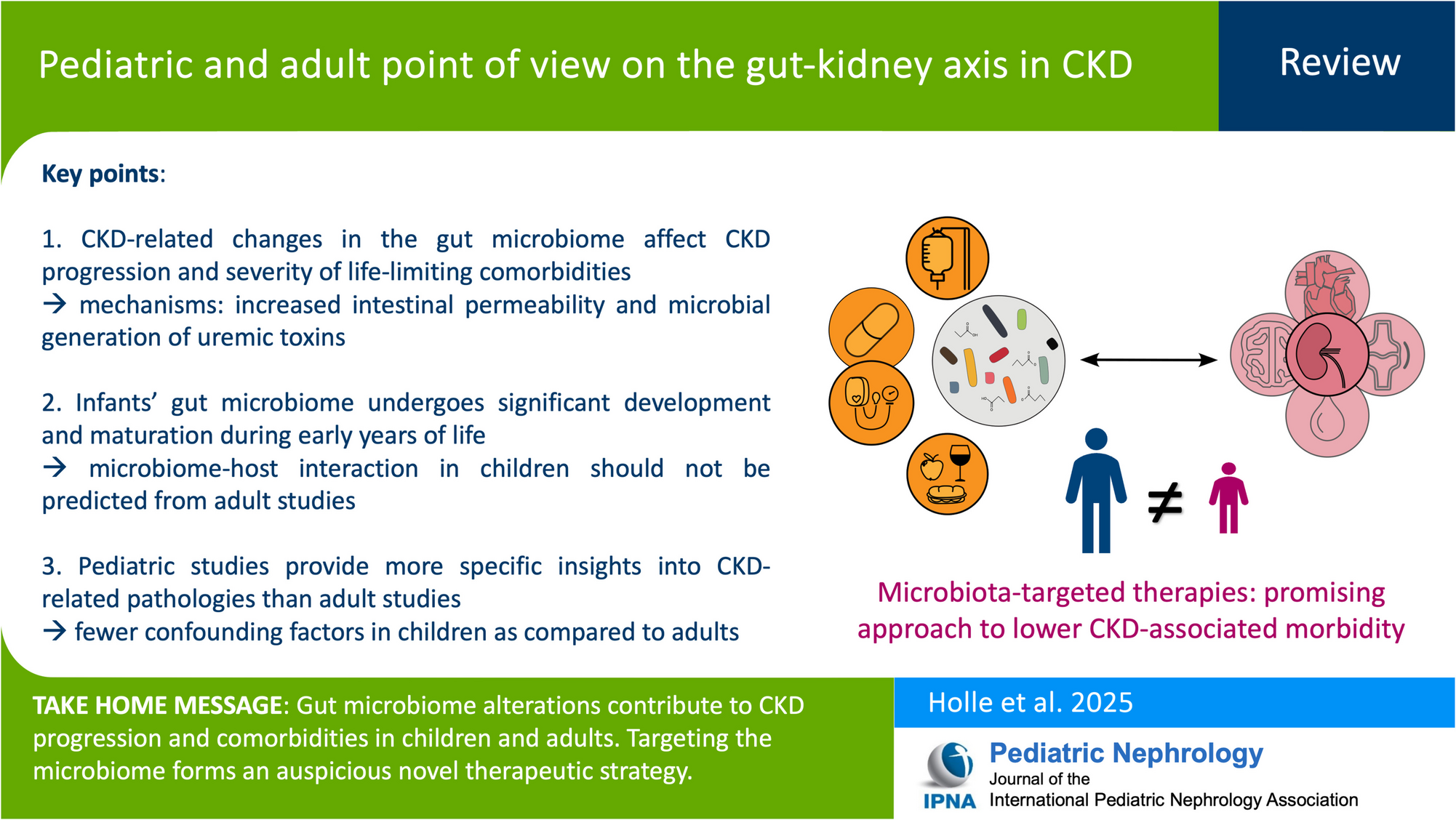

Supplement: Supplementary file 1 — Graphical abstract (PNG 981 KB) [file 467_2025_6780_MOESM1_ESM.png]
